# Supplementary material for: Valproic acid use is associated with diminished risk of contracting COVID-19, and diminished disease severity: Epidemiologic and in vitro analysis reveal mechanistic insights
Source: PLoS One. 2024 Aug 2;19(8):e0307154. doi: 10.1371/journal.pone.0307154 (PMC11296636; doi:10.1371/journal.pone.0307154)
Supplement: S1 Table — (PPTX) [file pone.0307154.s002.pptx]

## Slide 1
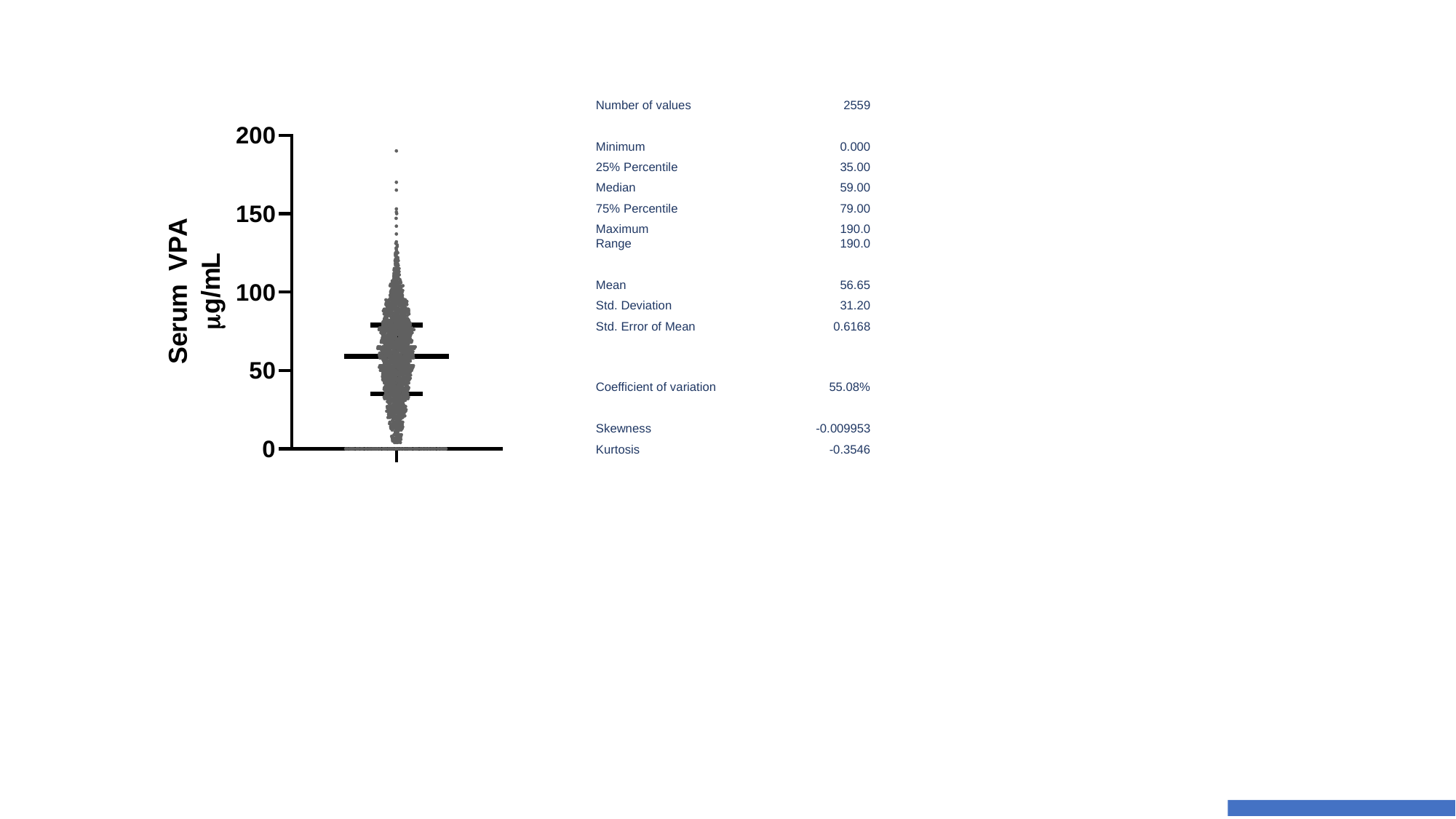

| Number of values | 2559 |
| --- | --- |
| | |
| Minimum | 0.000 |
| 25% Percentile | 35.00 |
| Median | 59.00 |
| 75% Percentile | 79.00 |
| Maximum | 190.0 |
| Range | 190.0 |
| | |
| Mean | 56.65 |
| Std. Deviation | 31.20 |
| Std. Error of Mean | 0.6168 |
| | |
| Coefficient of variation | 55.08% |
| | |
| Skewness | -0.009953 |
| Kurtosis | -0.3546 |
| | |

## Slide 2
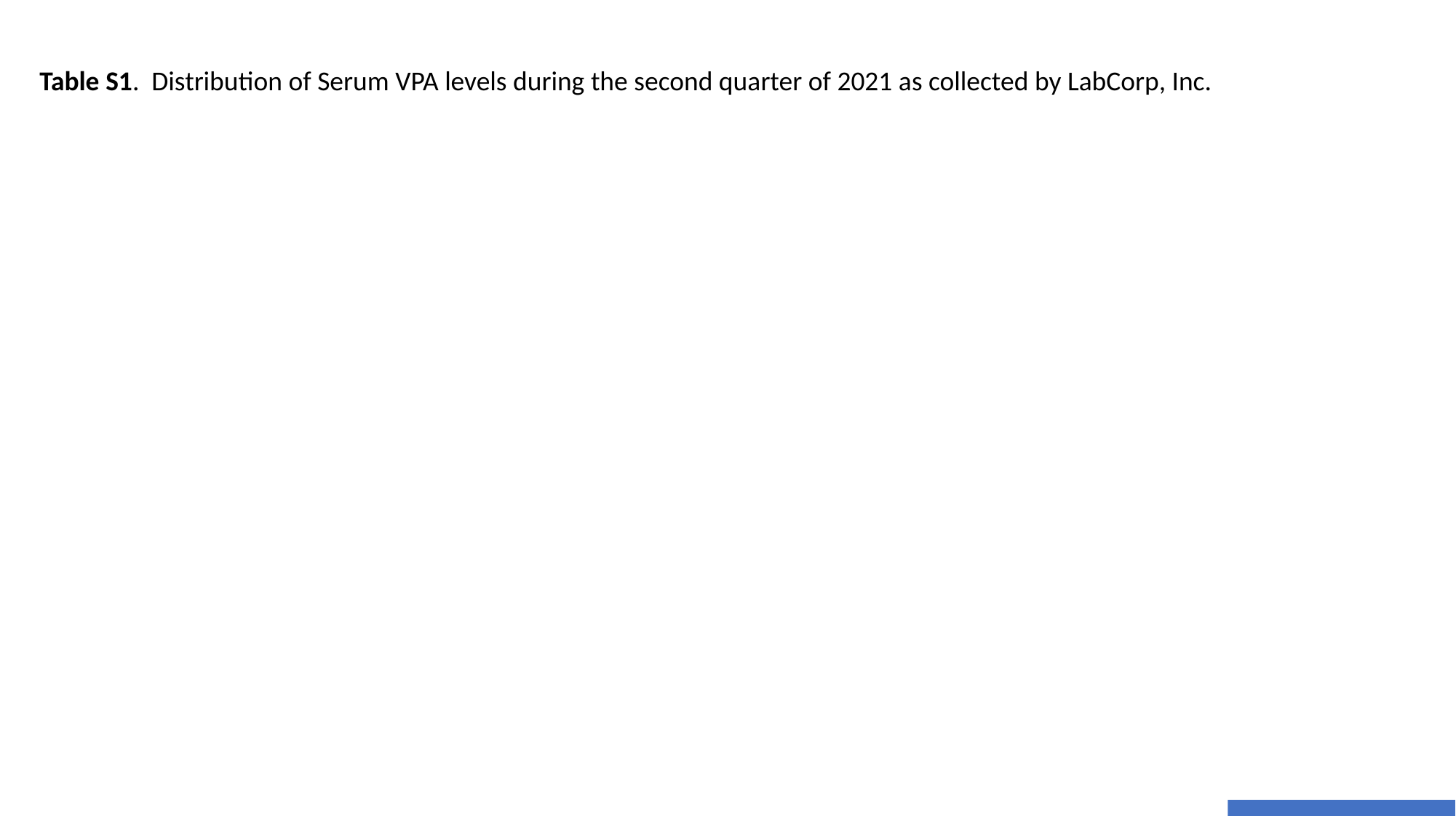

Table S1. Distribution of Serum VPA levels during the second quarter of 2021 as collected by LabCorp, Inc.
