# Supplementary material for: Valproic acid use is associated with diminished risk of contracting COVID-19, and diminished disease severity: Epidemiologic and in vitro analysis reveal mechanistic insights
Source: PLoS One. 2024 Aug 2;19(8):e0307154. doi: 10.1371/journal.pone.0307154 (PMC11296636; doi:10.1371/journal.pone.0307154)
Supplement: S2 Table — (PPTX) [file pone.0307154.s003.pptx]

## Slide 1
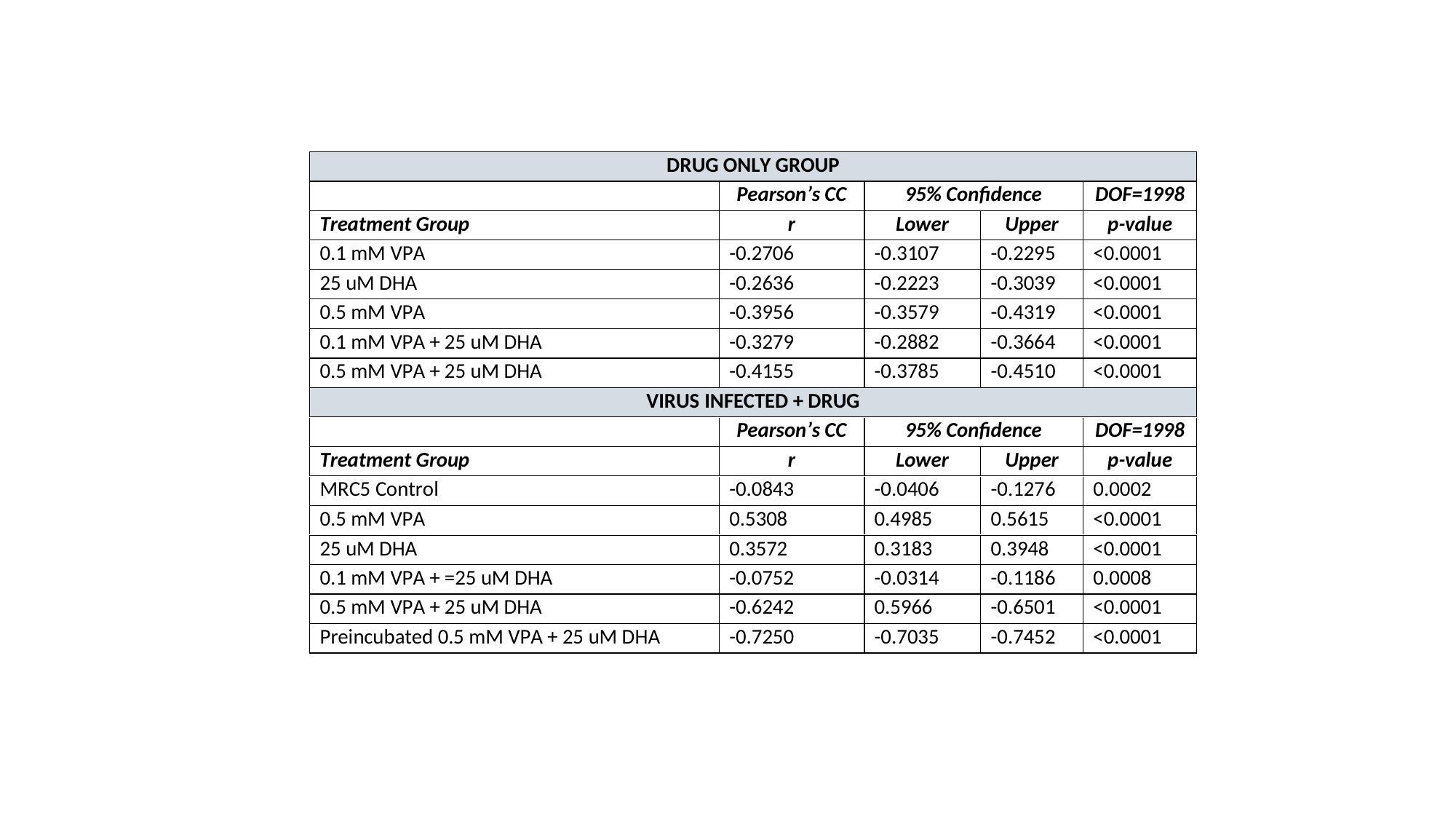

## Slide 2
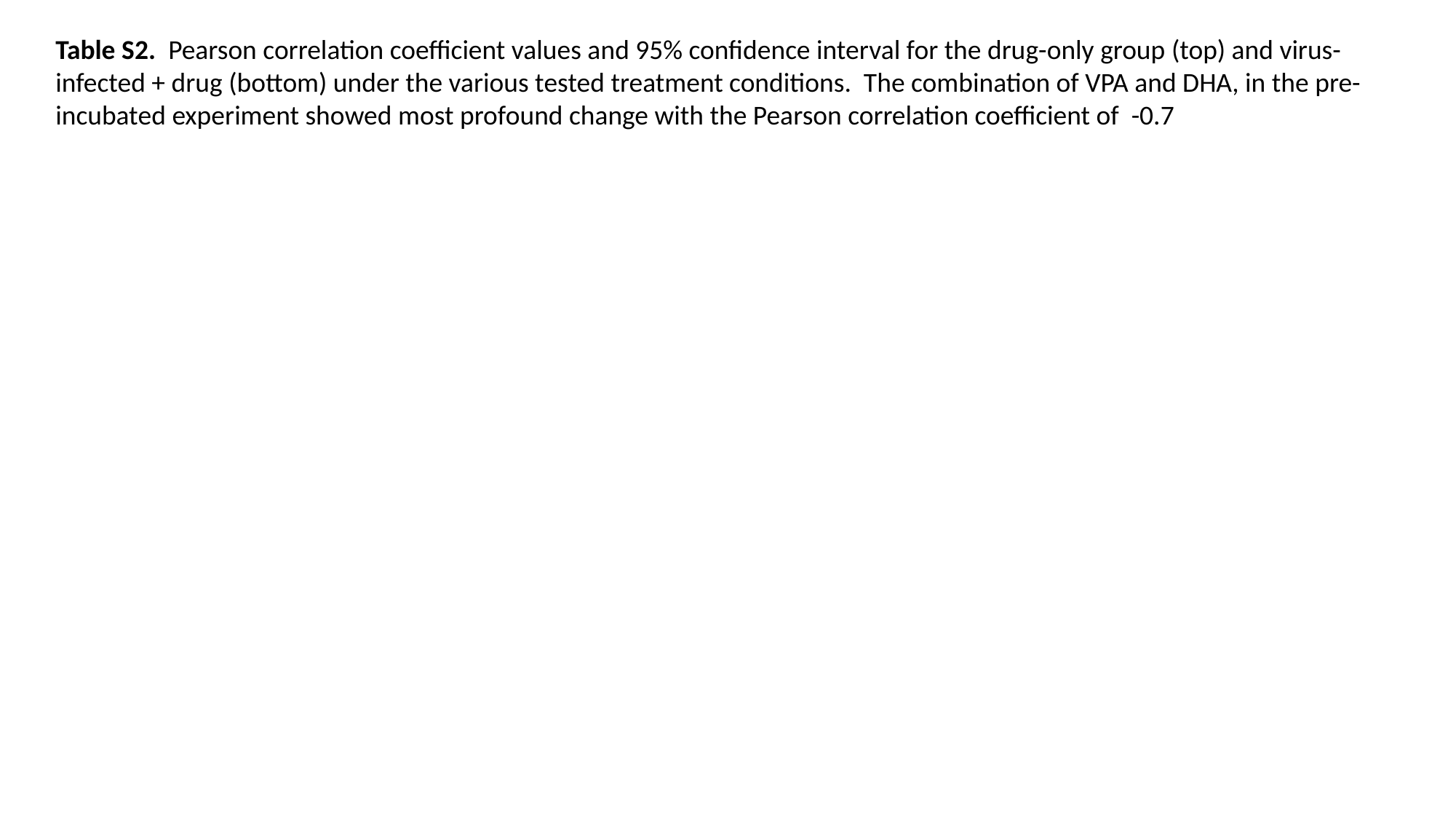

Table S2. Pearson correlation coefficient values and 95% confidence interval for the drug-only group (top) and virus-infected + drug (bottom) under the various tested treatment conditions. The combination of VPA and DHA, in the pre-incubated experiment showed most profound change with the Pearson correlation coefficient of -0.7
