# Supplementary material for: Valproic acid use is associated with diminished risk of contracting COVID-19, and diminished disease severity: Epidemiologic and in vitro analysis reveal mechanistic insights
Source: PLoS One. 2024 Aug 2;19(8):e0307154. doi: 10.1371/journal.pone.0307154 (PMC11296636; doi:10.1371/journal.pone.0307154)
Supplement: S3 Table — (PPTX) [file pone.0307154.s004.pptx]

## Slide 1
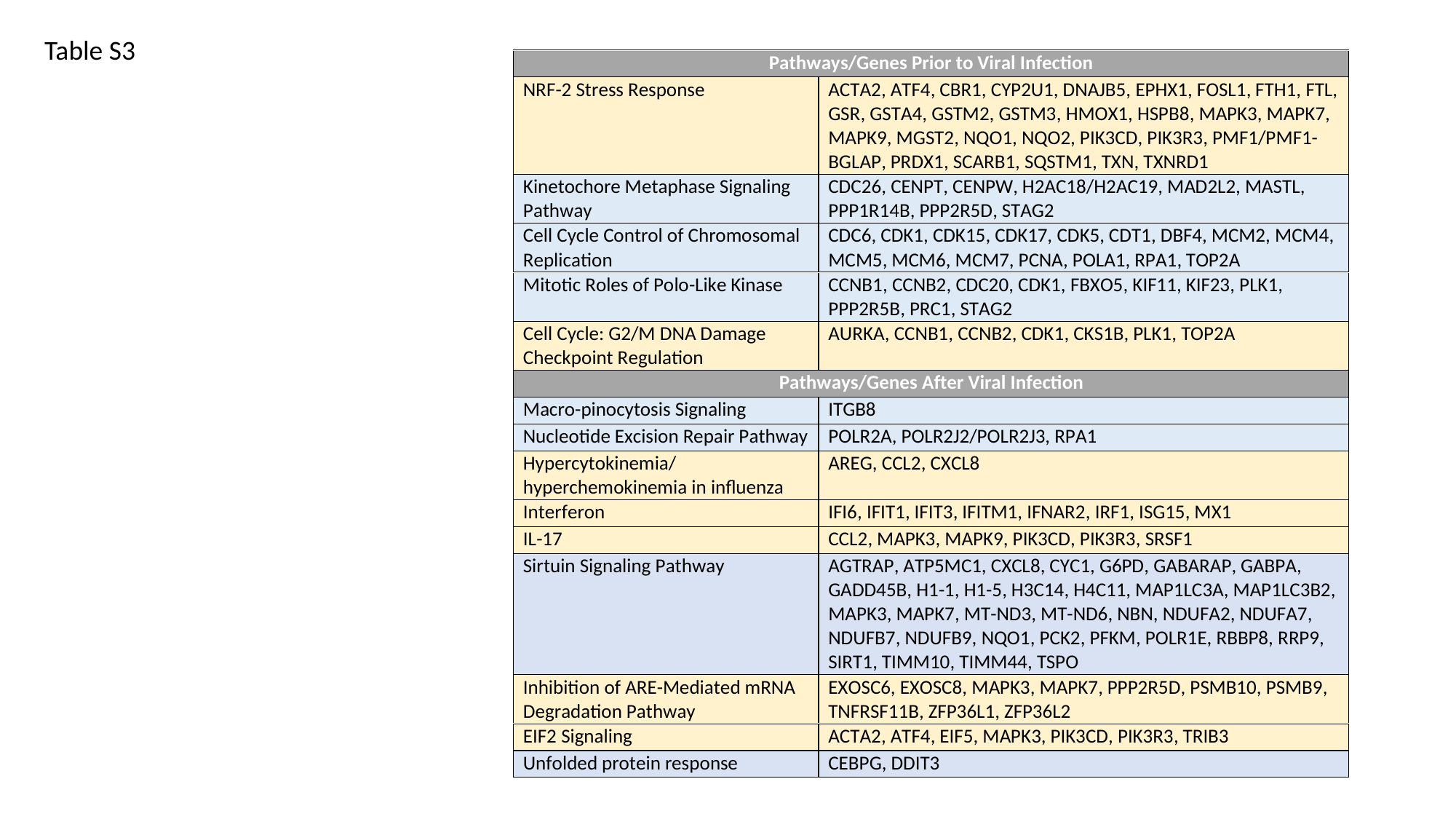

Table S3

## Slide 2
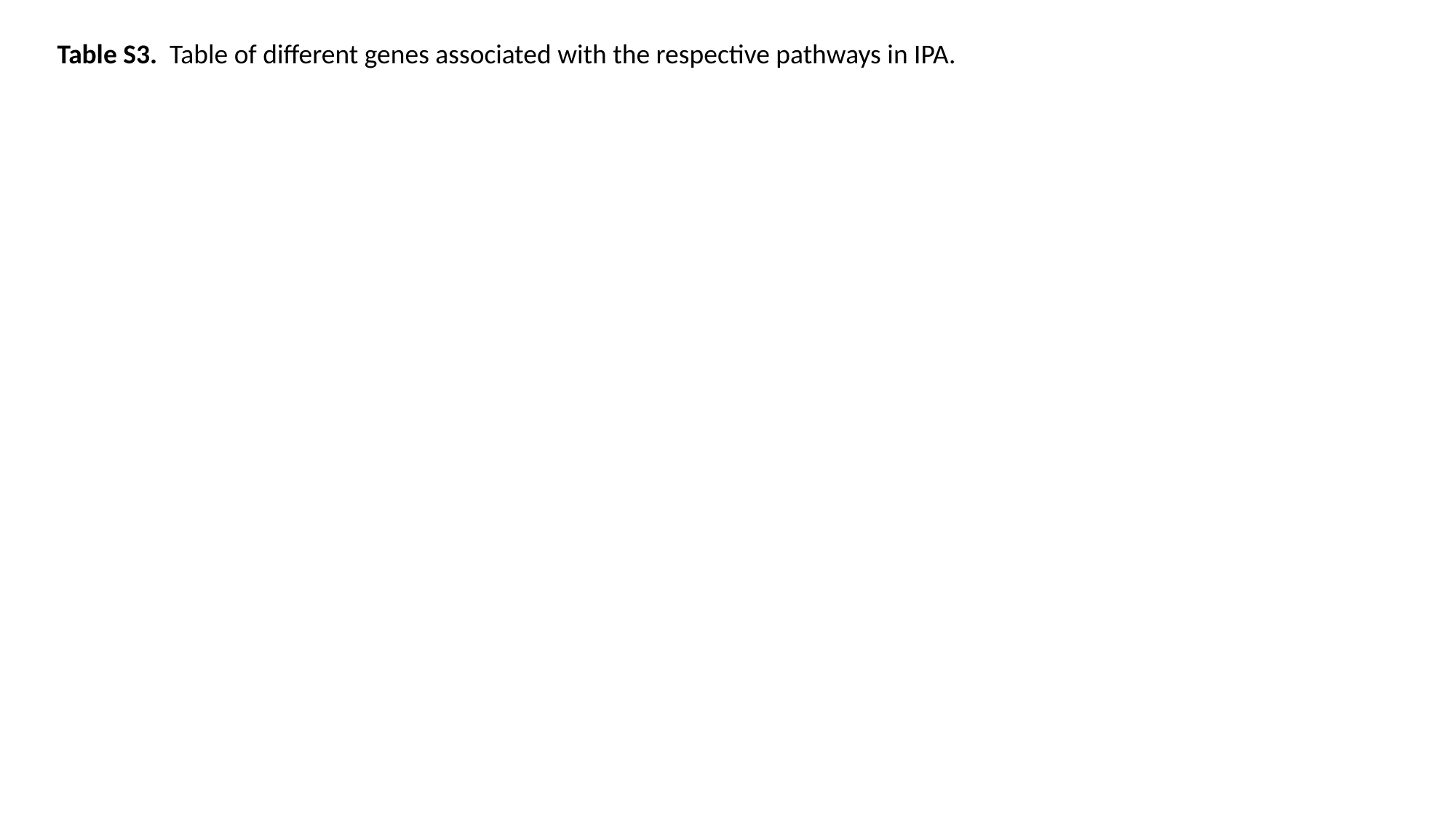

Table S3. Table of different genes associated with the respective pathways in IPA.
