# Supplementary material for: Valproic acid use is associated with diminished risk of contracting COVID-19, and diminished disease severity: Epidemiologic and in vitro analysis reveal mechanistic insights
Source: PLoS One. 2024 Aug 2;19(8):e0307154. doi: 10.1371/journal.pone.0307154 (PMC11296636; doi:10.1371/journal.pone.0307154)

24 HOURS : BRD2

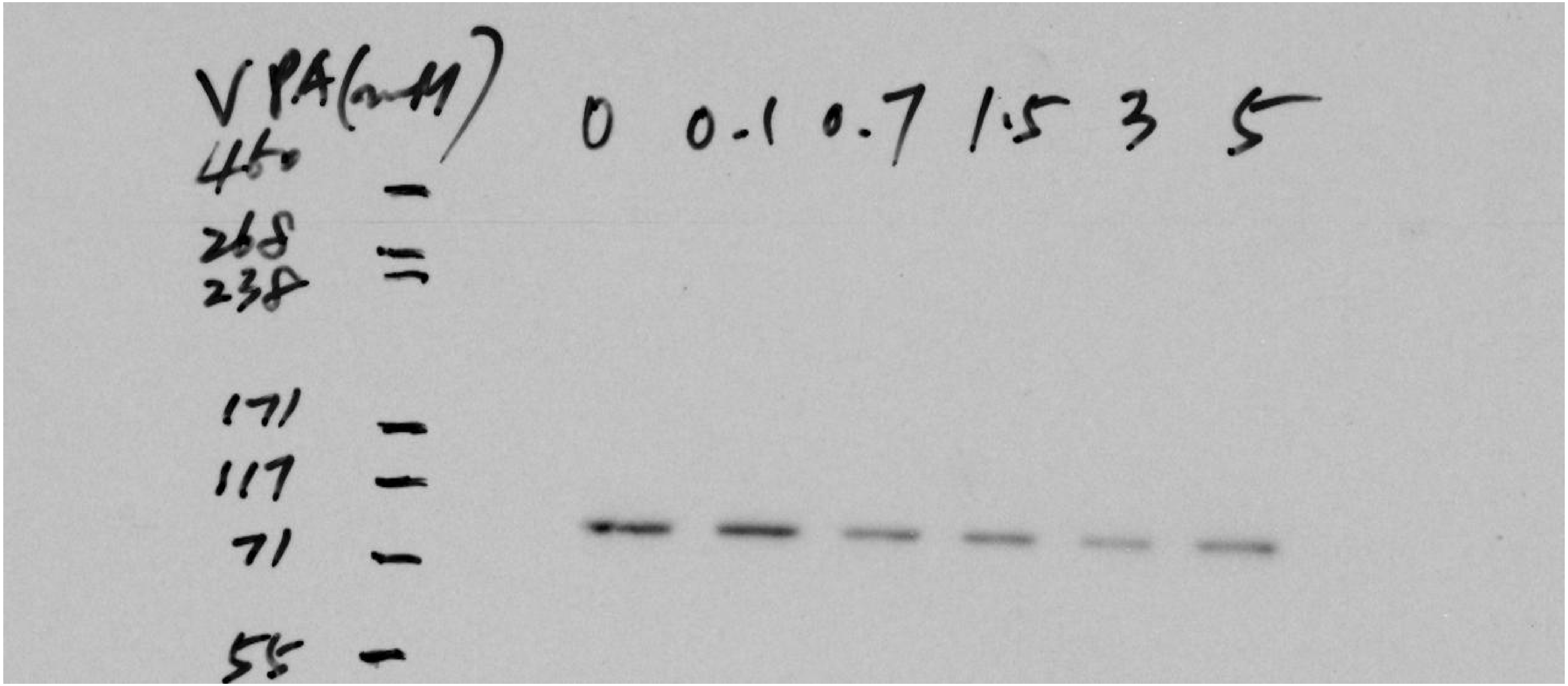

24 HOURS : DNMT1

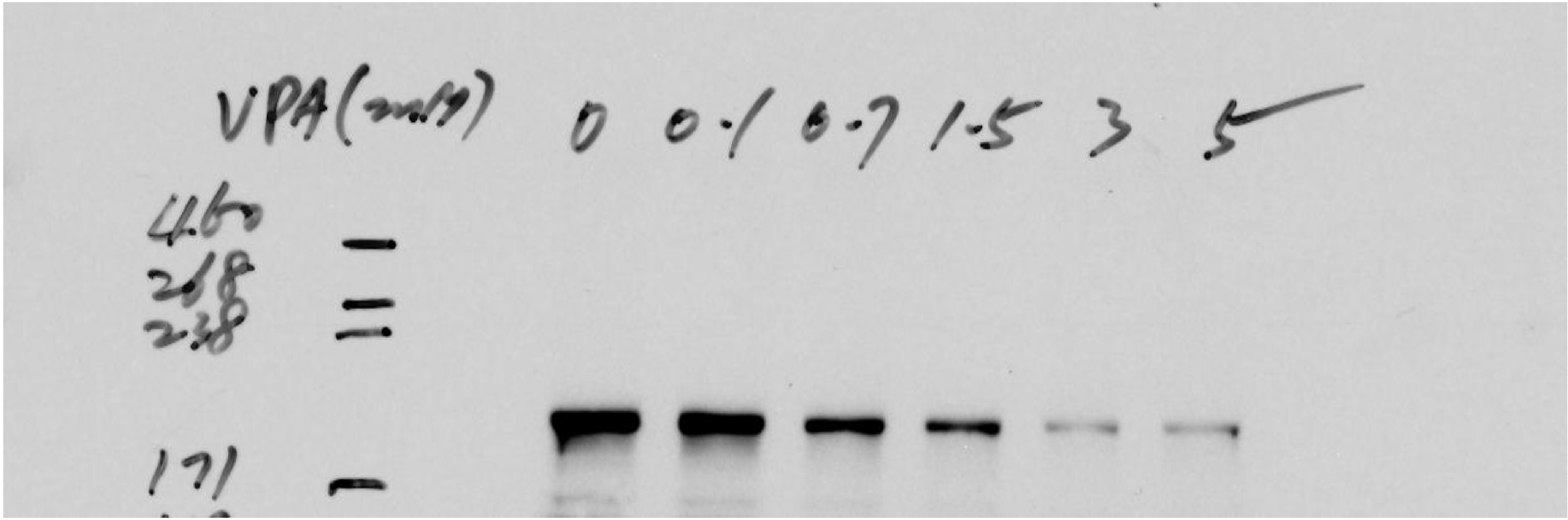

24 HOURS : GAPDH

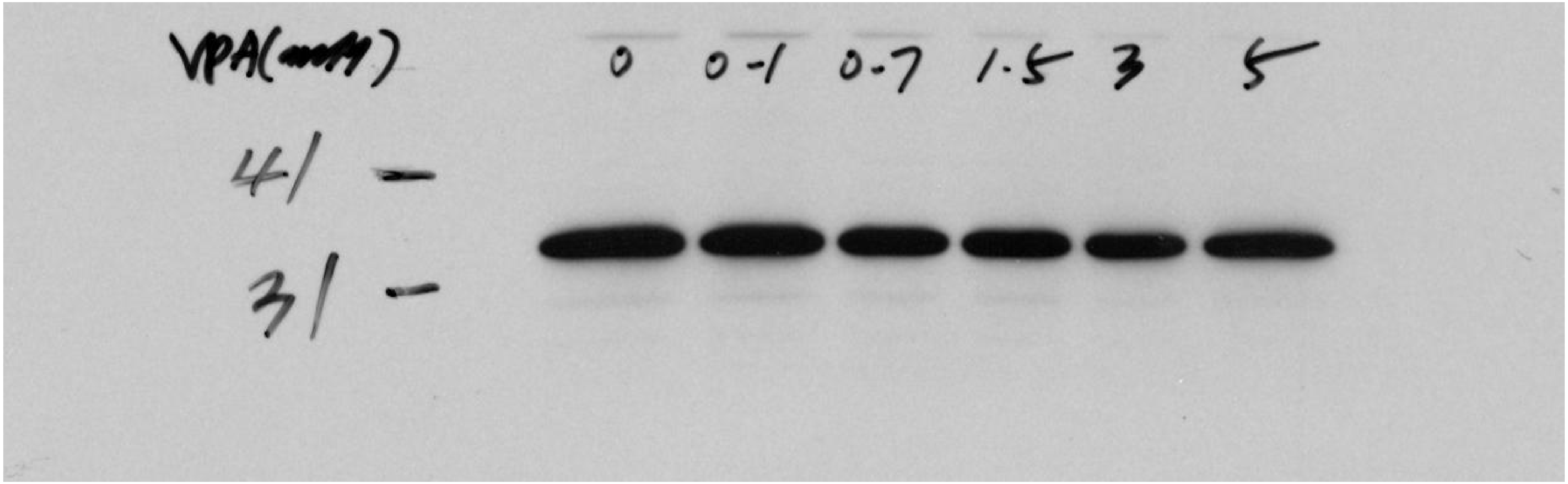

24 HOURS : HMOX1

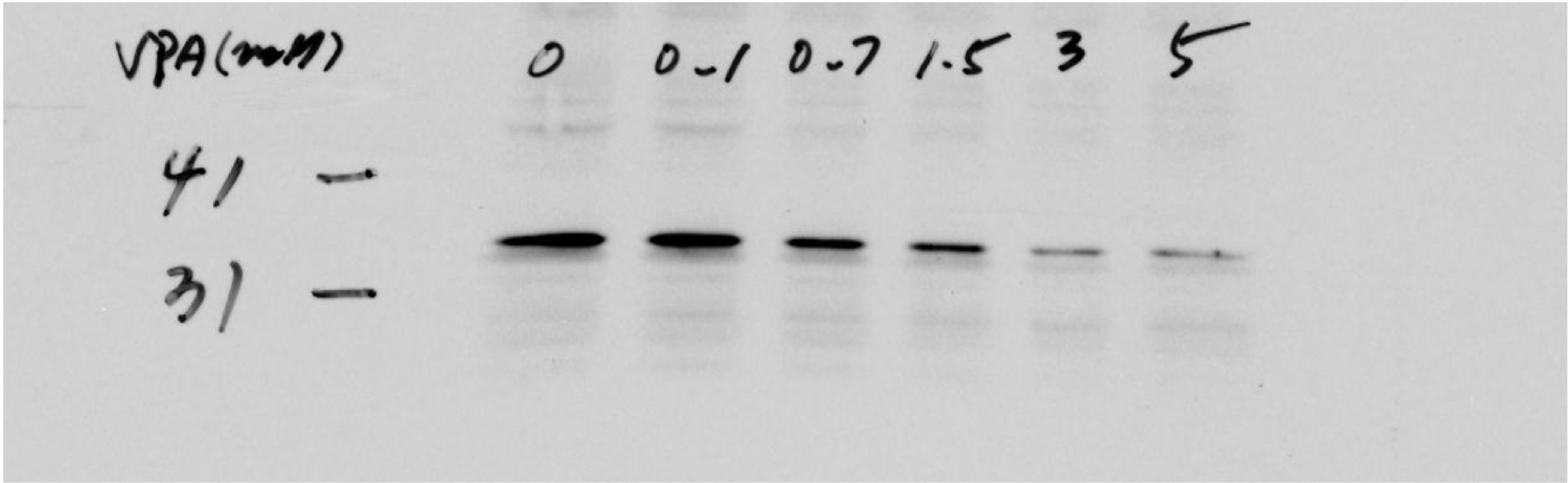

24 HOURS : PCNT

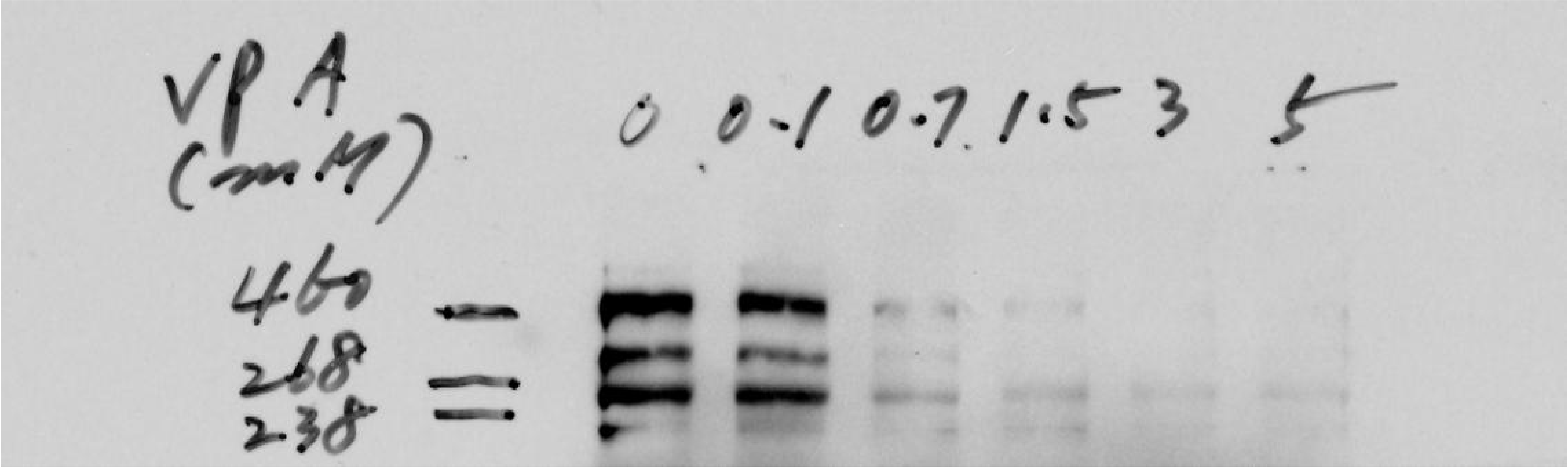

48 HOURS : BRD2

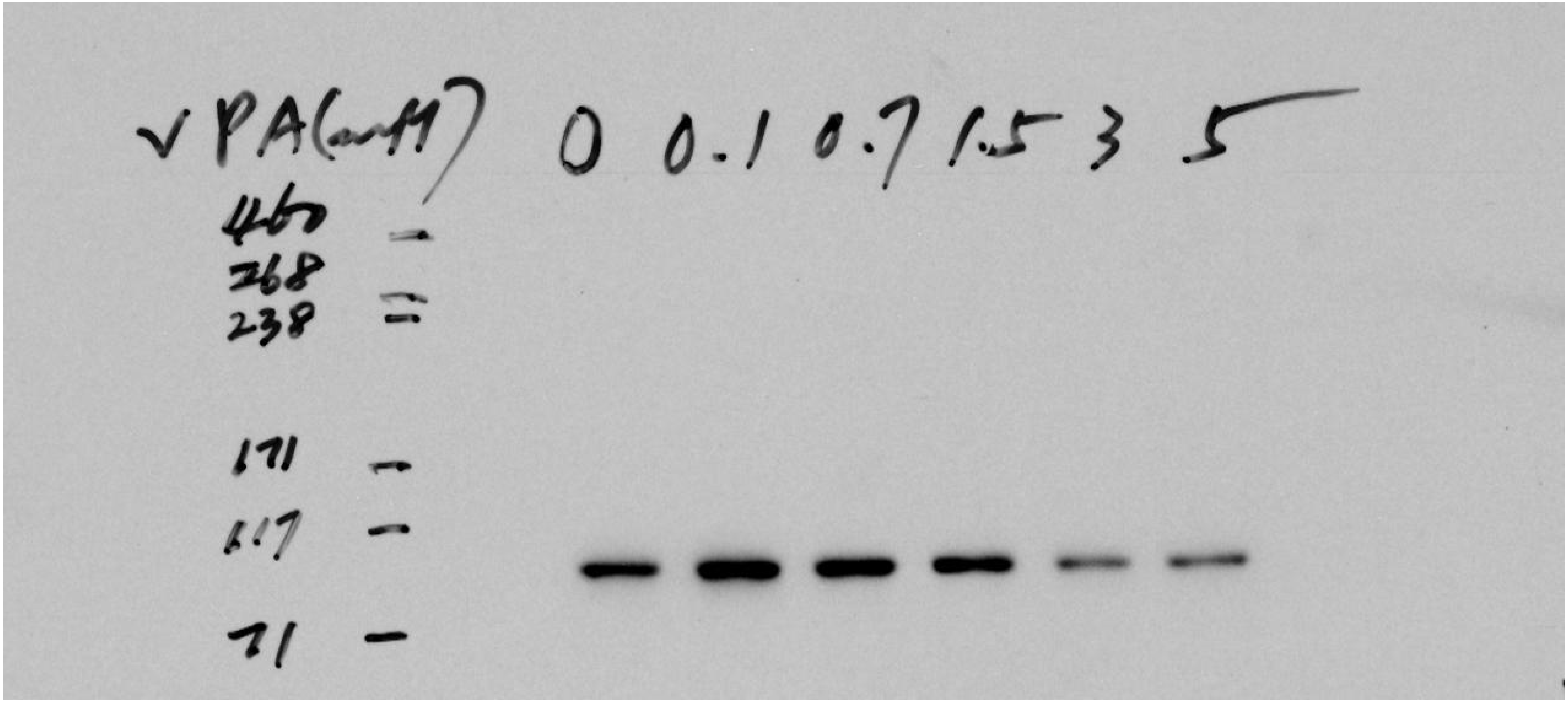

48 HOURS : DMNT1

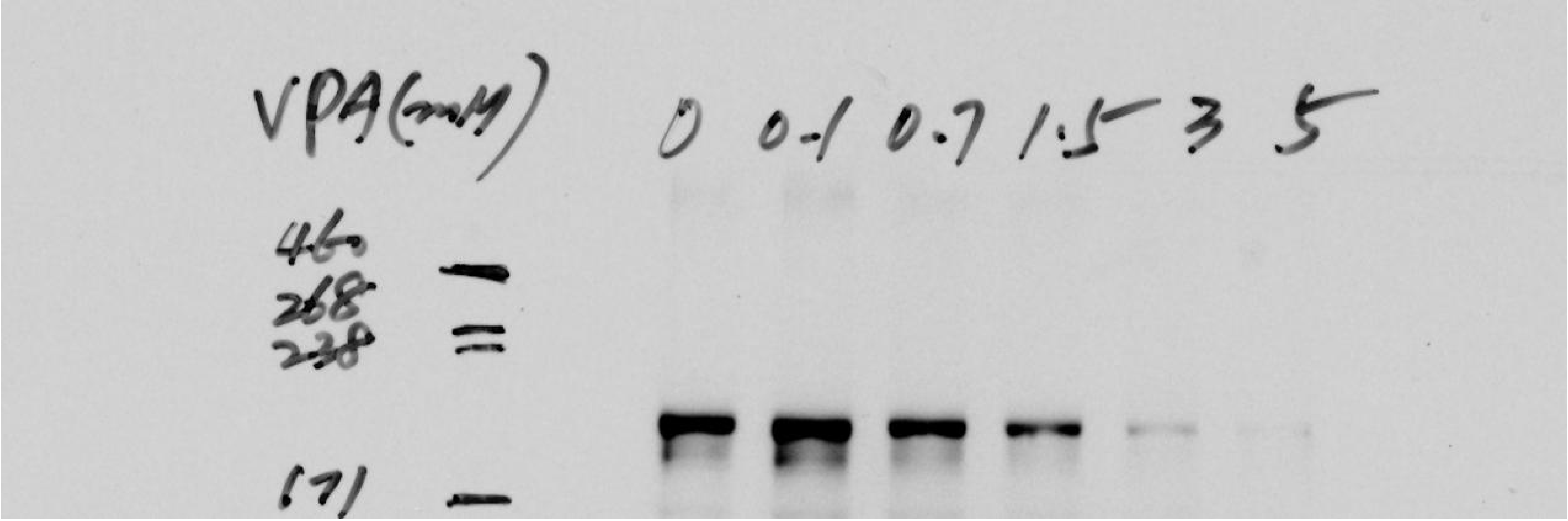

48 HOURS : GAPDH

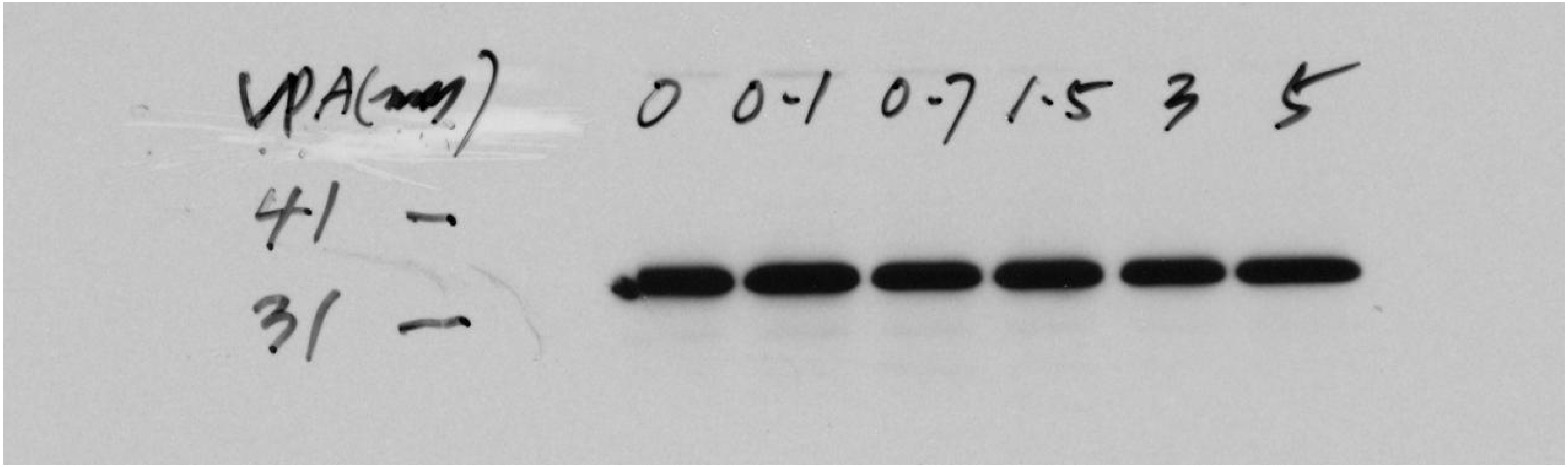

48 HOURS : HMOX1

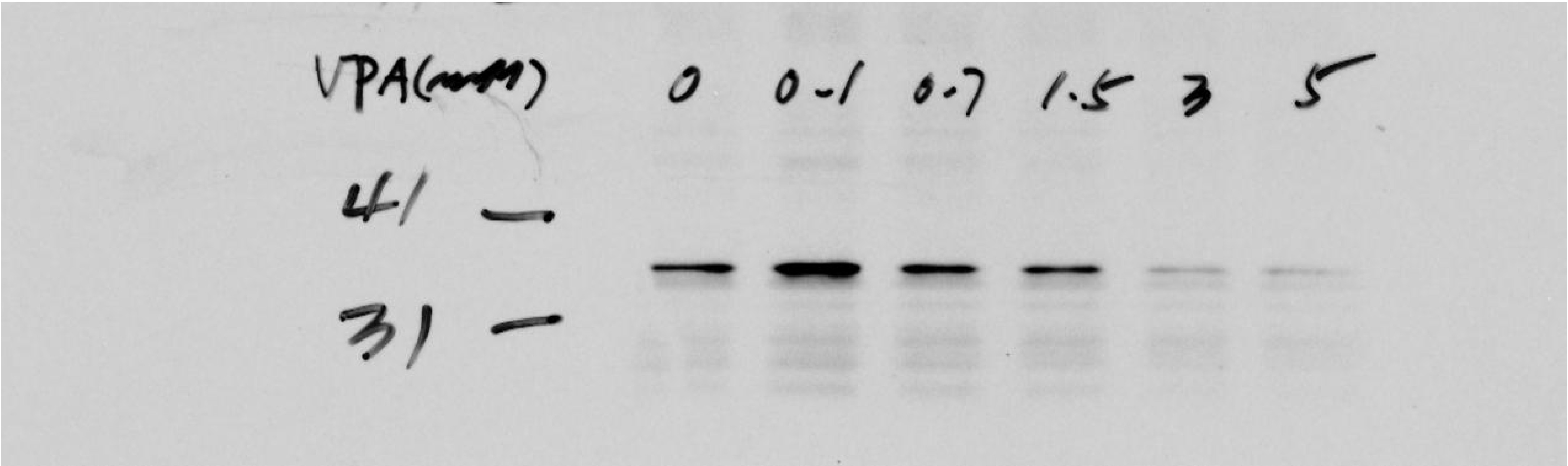

48 HOURS : PCNT

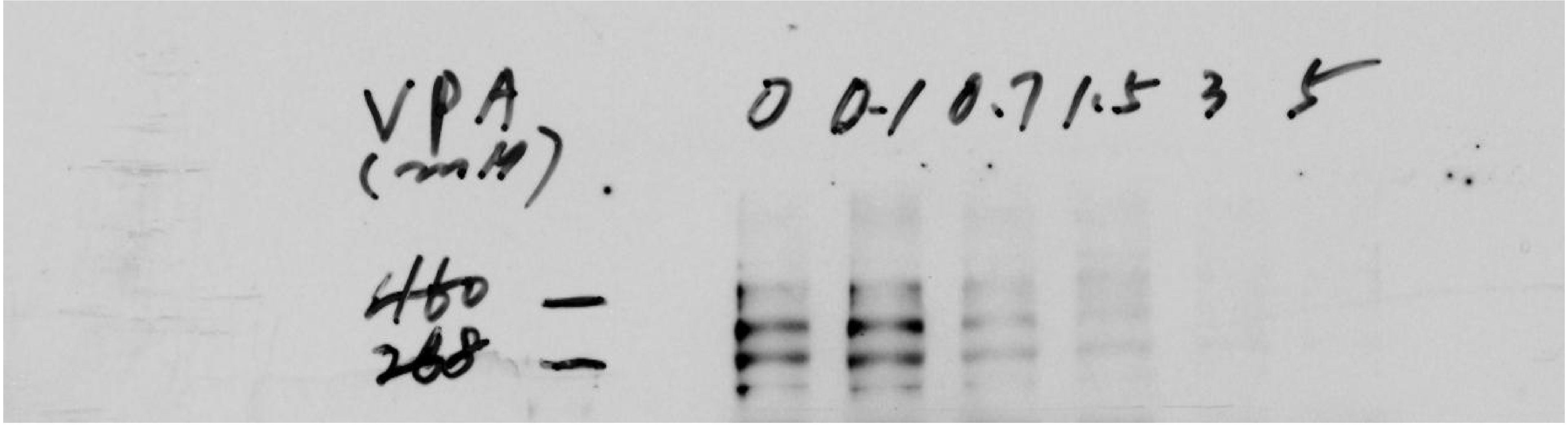

Supplement: S1 Raw images — (PDF) [file pone.0307154.s005.pdf]
