# Supplementary material for: Valproic acid use is associated with diminished risk of contracting COVID-19, and diminished disease severity: Epidemiologic and in vitro analysis reveal mechanistic insights
Source: PLoS One. 2024 Aug 2;19(8):e0307154. doi: 10.1371/journal.pone.0307154 (PMC11296636; doi:10.1371/journal.pone.0307154)
Supplement: Attachment — Submitted filename: Response to reviewers.pdf [file pone.0307154.s006.pdf]

UT Health San Antonio
School of Medicine

Department of Medical Education

PLOS-One
San Francisco, CA
plosone@plos.org.

Response to Editor's comments:
Re: Valproic acid Use is Associated with Diminished Risk of Contracting COVID-19, and Diminished Disease Severity: Epidemiologic and *in vitro* Analysis Reveal Mechanistic Insights

To the Editor:

Thank you for recognizing our work and giving us the opportunity to address the issues raised. We have addressed each point raised during the review process.

Editor Item #1:

We note you have included a table to which you do not refer in the text of your manuscript. Please ensure that you refer to Table 4 in your text; if accepted, production will need this reference to link the reader to the Table.

Response: Thank you for pointing it out. Now we have referenced the Table 4 in the main text (Line 424 , Page 22).

Editor Item #2:

We notice that your supplementary figures "Figure S1" are uploaded with the file type 'Figure'. Please amend the file type to 'Supporting Information'. Please ensure that each Supporting Information file has a legend listed in the manuscript after the references list."

Response: We have now uploaded Figure S1 to file type to 'Supporting Information'. Legend is listed in the manuscript after the reference list.

Item #1 Reviewer #1: *In the manuscript titled "Valproic acid Use is Associated with Diminished Risk of1 Contracting COVID-19, and Diminished Disease Severity:2 Epidemiologic and in vitro Analysis Reveal Mechanistic Insights", the authors attempted to provide understandings to use VPA as an antiviral agent against coronaviruses. The study is interesting and opens up the possibility of using VPA in combination with DHA as a potential drug to treat COVID. However, I have a few minor concerns.*

We thank reviewer1 for recognizing the potential of our research and encouraging comments. We have provided point-to-point response.

Figure S1B should be cited in the manuscript after the sentence (Line 444, page 24)
- **Response:** Thank you for pointing it out. Now, Figure S1B is cited as suggested.

Conclusion sentence for Figure 2 (Lines 476-478, page 25) may be rephrased for better understanding to the audience.

- **Response:** Thank you for the comment. The sentence is rephrased for the clarity.

I feel authors should provide higher resolution image for figures 2, 5C,5D and 6

- **Response:** We have provided higher resolution images for the figures.

Reviewer #2:
We thank Reviewer 2 for encouraging comments.

Sincerely,

Ron Rodriguez, MD., PhD.
